# Long-term risk of screen-detected and interval breast cancer after false-positive results at mammography screening: joint analysis of three national cohorts

**DOI:** 10.1038/s41416-018-0358-5

**Published:** 2018-12-19

**Authors:** Marta Román, Solveig Hofvind, My von Euler-Chelpin, Xavier Castells

**Affiliations:** 1 Department of Epidemiology and Evaluation, IMIM (Hospital del Mar Medical Research Institute), Barcelona, Spain; 2 Network on Health Services in Chronic Diseases (REDISSEC), Barcelona, Spain; 3 Department of Screening, Cancer Registry of Norway, Oslo, Norway; 4 Oslo and Akershus University College of Applied Sciences, Faculty of Health Science, Oslo, Norway; 5 Department of Public Health, University of Copenhagen, Copenhagen, Denmark

**Keywords:** Breast cancer, Cancer epidemiology

## Abstract

**Background:**

We assessed the long-term risk of screen-detected and interval breast cancer in women with a first or second false-positive screening result.

**Methods:**

Joint analysis had been performed using individual-level data from three population-based screening programs in Europe (Copenhagen in Denmark, Norway, and Spain). Overall, 75,513 screened women aged 50–69 years from Denmark (1991–2010), 556,640 from Norway (1996–2008), and 517,314 from Spain (1994–2010) were included. We used partly conditional Cox hazards models to assess the association between false-positive results and the risk of subsequent screen-detected and interval cancer.

**Results:**

During follow-up, 1,149,467 women underwent 3,510,450 screening exams, and 10,623 screen-detected and 5700 interval cancers were diagnosed. Compared to women with negative tests, those with false-positive results had a two-fold risk of screen-detected (HR = 2.04, 95% CI: 1.93–2.16) and interval cancer (HR = 2.18, 95% CI: 2.02–2.34). Women with a second false-positive result had over a four-fold risk of screen-detected and interval cancer (HR = 4.71, 95% CI: 3.81–5.83 and HR = 4.22, 95% CI: 3.27–5.46, respectively). Women remained at an elevated risk for 12 years after the false-positive result.

**Conclusions:**

Women with prior false-positive results had an increased risk of screen-detected and interval cancer for over a decade. This information should be considered to design personalised screening strategies based on individual risk.

## Introduction

Women with abnormal mammographic findings at screening are recalled for further assessment, including additional imaging and eventually needle biopsy. If no breast cancer is diagnosed, the screening result is considered as false-positive, which may cause distress and anxiety to the women.^1–3^ Studies have shown 20% cumulative risk of a false-positive result for women who start screening at age 50 and undergo 10 biennial screens.^4,5^ False-positive screening results have been associated with an increased risk of breast cancer.^6–11^ The risk has been shown to remain elevated more than 6 years after the false-positive screening result, suggesting a biological susceptibility for these women to develop a breast cancer later in life.^6,9,11^

Further assessments that turn out negative might represent misclassification and missed cancers,^12,13^ i.e., women recalled for further assessments were declared negative, when they should actually have been declared as having cancer. Misclassified cancers would emerge mostly in a short time interval after the screening-negative result, either as an interval breast cancer during the following 24 months, or as a screen-detected cancer at 2 years. However, there is limited evidence regarding the long-term risk of interval breast cancer and screen-detected cancer after a false-positive result. We hypothesise that if the long-term increase in risk is similar for screen-detected cancer and for interval breast cancer in women with false-positive results, it suggests that false-positives may be risk markers for breast cancer development later in life rather than precursors of malignancy.

Given that false-positive results are the most common adverse effect of screening mammography, the risk attributable to false-positive results examinations could be substantial if the association with breast cancer is strong. Women with false-positive screening results represent a group of women with potential for stratification based on their individual breast cancer risk. Although few women experience a false-positive screening result more than once during their lifespan, on the longitudinal perspective of sequential biennial screening examinations for each woman, their risk of breast cancer should be assessed. No previous study has examined whether a second false-positive screening result influences the risk of breast cancer to a greater extent, or its impact on the risk of an interval breast cancer.

Using individual level data from long standing mammography programs in Copenhagen in Denmark, Norway, and Spain, we conducted a joint study to evaluate the association between a first and second false-positive screening result and the long-term risk of interval breast cancer and screen-detected cancer.

## Methods

### Study population and data sources

We performed a joint analysis using individual level data from the population-based screening programs in the Copenhagen Mammography Register in Denmark, BreastScreen Norway, and the Spanish Breast Cancer Screening Program. All three screening programs target women aged 50–69 years, perform biennial screening, and are run according to the European Guidelines for Quality Assurance in Breast Cancer Screening.^14^ The programs have been described in detail elsewhere.^11,15–19^

Briefly, the organised and population-based screening program in the municipality of Copenhagen started in 1991, and there was a national roll-out of the program in 2008–2010. Two trained breast radiologists independently interpret the screening mammograms and classify the women as negative or positive. In case of disagreement a third radiologist is consulted, until consensus is reached. Women classified as positive are recalled for additional assessment. Ninety-five percent of recall examinations take place within 10 working days after mammographic reading. Women with no breast cancer diagnosed after additional assessment are referred back to routine screening, while women diagnosed with breast cancer are referred for treatment.

BreastScreen Norway started as a pilot in 1996 and became nationwide in 2005.^17^ Two trained breast radiologists read the screening mammograms independently and give a score 1–5 for each breast. Cases with a score of 2 or higher by one or both radiologists are discussed at a consensus meeting where the final decision about recall is taken. Recall examinations take place 1–2 weeks after the screening examination. If no malignancy is stated, the women are referred back to screening. All women diagnosed with breast cancer are referred to treatment.

Population-based screening in Spain started in one setting in 1990 and became nationwide in 2006.^16^ We included data from eight centres of the mammography screening program in Spain. The screening mammograms are interpreted and classified according to the BI-RADS scale or equivalent^15^ by two trained breast radiologists. Women with abnormal mammographic findings are recalled for further assessments within 3 weeks after mammographic reading. If no malignancy is stated, women are referred back to screening, while women diagnosed with breast cancer are referred for treatment.

Our study included information about 75,513 women screened in Denmark, (1991–2010), 556,640 screened in Norway (1996–2008), and 517,314 screened in Spain (1994–2010) (Supplementary material, section 1). Overall, the women contributed 3,510,450 screening exams during the study period. Further, the women were additionally followed-up for 2 years at the end of the study period for interval breast cancer diagnosis. Interval cancers were identified by linking nationwide cancer registries to screening data in Denmark and Norway. In Spain, since there is no nationwide cancer registry, screening registry data were linked to population-based registries (regional Minimum Basic Data Set, regional cancer registries), and hospital-based cancer registries, which ensured a high compliance of interval breast cancer cases.

### Measures and definitions

A false-positive screening result was defined as a recall for further assessment without confirmation of cancer diagnosis, regardless of the procedures performed (additional imaging and/or invasive procedures with benign outcome). A screen-detected cancer was defined as a breast cancer (ductal carcinoma in situ or invasive cancer) diagnosed at further assessment due to abnormal findings of screening mammography interpretation.

An interval breast cancer was defined as a breast cancer diagnosed after a negative screening or after false-positive screening, either before the next screening (2 years) or within 2 years after the final screening among women who reach the upper age limit. A woman was considered an irregular attendee if she re-attended screening after having missed at least one biennial screening invitation. In addition, information was collected on whether the screening examination was performed with screen film mammography (SFM) or full field digital mammography (FFDM).

For each woman, person-years at risk were calculated from the date of first screen. Women contributed person-years at risk to the screened negative group from the date of first screen until the date of first false-positive result, if any. Contribution to the first false-positive group started at the date of first false-positive result until the date of second false-positive result, if any, whereupon they started contributing to the second false-positive group. Censoring times during follow-up were calculated differently for the analyses of screen-detected and interval breast cancers. To calculate the risk of screen-detected cancer, women were censored at the date of last screening examination in the study period, or at the date of breast cancer diagnosis following last screening participation. Since interval breast cancers can show up at any time in the two-year period after a negative screening examination, to calculate the risk of interval breast cancer women were censored at 24 months after last recorded screening date in the study period, or at the date of interval cancer diagnosis, whichever came first. Women with a screen-detected cancer diagnosed at first screen were not included in the analyses because they could not have a previous negative or false-positive result.

### Statistical analysis

We compared characteristics of the study population in the three countries. We examined the crude rates of screen-detected and interval breast cancers overall and stratified by country. Rates were calculated as the number of screen-detected cancers and number of interval cancers, divided by the number of women-years at risk in each group. Confidence intervals for rates were calculated using exact Poisson distribution.

We used a partly conditional frailty Cox proportional hazard model to assess the association between a false-positive screening result (first or second false-positive result separately) and the risk of breast cancer.^20^ By using a partly conditional Cox model, we included all screening mammograms received by an individual woman accounting for within woman correlation. Since the information is included at a mammogram level, the covariates in the models were analyzed as time-changing variables. Separate models were used to estimate the risk of screen-detected and interval breast cancer. To account for possible confounders, all models were adjusted for age at screen (continuous), type of attendance (regular or irregular), and mammography type (SFM or FFDM). The individual effect of the adjusting variables in the estimates is presented in Supplementary materials, section 4. A country-specific random effect was also included in the models as a frailty component to account for the unobserved heterogeneity among screening tests performed in the same program (Denmark, Norway, and Spain). We estimated the hazard ratios (HR) and 95% confidence intervals (95% CI) of screen-detected and interval breast cancer from the regression models. Results of models by screening mammogram result are presented graphically in the form of model-based adjusted survival curves. The proportional hazards assumption was ascertained by assessment of log–log survival plots. Also, we tested for an interaction between false-positive screening results and log time. This test found no evidence of violation of the proportional hazards assumption between the covariate and time. We performed two sensitivity analyses. First, we tested the impact in the estimates of the heterogeneity across countries by testing the final adjusted model excluding the country effect, including country as a fixed factor (fixed effects model), and as a random effect in the final adjusted model. Secondly, we tested the impact of misclassification on our estimates by excluding from the analyses screen-detected cancers and interval breast cancers diagnosed in the first 26 months of follow-up. All tests were two-sided with a 5% significance level. Statistical analyses were conducted in R 3.4.2 (R Foundation for Statistical Computing, Austria).

## Results

We analyzed data from 1,149,467 women aged 50–69 years at screen. The average follow-up for screen-detected cancers was 5.6 years, while for interval breast cancers it was 6.3 years. During follow-up, 10,623 women were diagnosed with a screen-detected breast cancer, 5700 women with interval breast cancer, 113,634 women had a first false-positive screening result, and 8149 had a second false-positive screening result (Supplementary material, section 1). The highest proportion of first false-positive results occurred among women aged 50–54, while second false-positive results were more frequent among women aged 55–59 years (Table 1).

**Table 1:** Characteristics of the study population by screening mammogram result. Women screened age 50–69 years

	Mammogram classification		
	First FP result, *N* = 113,634	Second FP result, *N* = 8149	True negative, *N* = 3,388,667
Age at mammography, mean (SD)	56.4 (5.6)	59.3 (5.1)	58.3 (5.8)
Age at mammography, 5 years, *N* (%)			
50–54	52,460 (46.2%)	1822 (22.4%)	1,025,119 (30.3%)
55–59	26,919 (23.7%)	2571 (31.5%)	963,718 (28.4%)
60–64	21,191 (18.6%)	2149 (26.4%)	834,492 (24.6%)
65–69	13,064 (11.5%)	1607 (19.7%)	565,338 (16.7%)
Mammography type, *N* (%)			
SFM	88,181 (77.6%)	5445 (66.8%)	2,744,952 (81.0%)
FFDM	25,453 (22.4%)	2704 (33.2%)	643,715 (19.0%)
Type of attendance, *N* (%)			
Prevalent	64,073 (56.4%)	0 (0.0%)	1,085,394 (32.0%)
Subsequent regular	46,684 (41.1%)	7453 (91.5%)	2,204,537 (65.1%)
Subsequent irregular	2877 (2.5%)	696 (8.5%)	98,736 (2.9%)

*FP* false-positive

Breast cancer rates stratified by country showed that in all three countries, the highest crude rates of screen-detected and interval breast cancer were found in women with a second false-positive result, and the lowest rates among those with negative tests (Table 2). Across countries, Spain had the lowest crude rates of screen-detected and interval breast cancer compared with Denmark and Norway. For women with a false-positive result, the rates of screen-detected cancer per 1000 women-years ranged from 2.84 (95% CI: 2.60–3.08) in Spain to 4.72 (95% CI: 3.93–5.52) in Denmark. The rates of interval breast cancer ranged from 1.06 (95% CI: 0.95–1.18) in Spain to 1.92 (95% CI: 1.73–2.10) in Norway.

**Table 2:** Rates of screen-detected cancer and interval breast cancer by screening mammogram result and country

	Screen detected cancer			Interval breast cancer		
	Women-years at risk	Number of cases	Breast cancer rate (95% CI) per 1000 women-years (‰)	Women-years at risk	Number of cases	Breast cancer rate (95% CI) per 1000 women-years (‰)
Denmark						
Negative test	358,686	983	2.74 (2.57–2.91)	494,348	558	1.13 (1.04–1.22)
False-positive result	28,797	136	4.72 (3.93–5.52)	39,571	73	1.84 (1.42–2.27)
Second false-positive result	805	9	11.18 (3.88–18.49)	1189	3	2.52 (0.00–5.38)
Norway						
Negative test	2,177,166	4992	2.29 (2.23–2.36)	3,181,417	2561	0.80 (0.77–0.84)
False-positive result	140,541	629	4.48 (4.13–4.83)	223,411	428	1.92 (1.73–2.10)
Second false-positive result	4780	33	6.90 (4.55–9.26)	9425	20	2.12 (1.19–3.05)
Spain						
Negative test	2,059,408	3254	1.58 (1.53–1.63)	2,955,223	1696	0.57 (0.55–0.60)
False-positive result	191,101	543	2.84 (2.60–3.08)	306,352	325	1.06 (0.95–1.18)
Second false-positive result	9602	44	4.58 (3.23–5.94)	19,458	36	1.85 (1.25–2.45)

The overall rates of screen-detected cancer and interval breast cancer by screening mammogram result are presented in Table 3. For those with a true-negative result, the rate of screen-detected cancer per 1000 women-years at risk was 2.01 (95% CI: 1.97–2.05). Among those with a first false-positive result the rate per 1000 women-years was 3.63 (95% CI: 3.43–3.83), whereas among those with a second false-positive result the rate was 5.66 (95% CI: 4.47–6.86). The overall rate of interval breast cancer per 1000 women-years was 0.73 (95% CI: 0.71–0.75) for negative tests, 1.45 (95% CI: 1.35–1.55) for a false-positive result, and 1.96 (95% CI: 1.46–2.46) for a second false-positive result.

**Table 3:** Overall rates of screen detected cancer and interval cancer by screening mammogram result

	Women-years at risk	Number of cases	Breast cancer rate (95% CI) per 1000 women-years (‰)
Screen-detected cancer			
Negative test	4,595,260	9229	2.01 (1.97–2.05)
False-positive result	360,439	1308	3.63 (3.43–3.83)
Second false-positive result	15,186	86	5.66 (4.47–6.86)
Interval breast cancer			
Negative test	6,630,989	4815	0.73 (0.71–0.75)
False-positive result	569,334	826	1.45 (1.35–1.55)
Second false-positive result	30,072	59	1.96 (1.46–2.46)

The adjusted Cox proportional hazards models analyses showed that women with false-positive results were at an increased risk of screen-detected cancer and interval breast cancer compared with women with negative tests (Table 4). Compared to women with negative test, women with a first false-positive result had double the risk of screen-detected cancer (HR = 2.04, 95% CI: 1.93–2.16) and interval breast cancer (HR = 2.18, 95% CI: 2.02–2.34), whereas women with a second false-positive result had over a four-fold risk of screen-detected and interval breast cancer (HR = 4.71, 95% CI: 3.81–5.83 and HR = 4.22, 95% CI: 3.27–5.46, respectively).

**Table 4:** Adjusted and unadjusted hazard ratios (HR) of screen-detected cancer and interval breast cancer for women with false-positive screening results compared to women with negative results

	Women-years at risk	Number of cases	Unadjusted HR (95% CI)	Adjusted HR (95% CI)^a^
Screen-detected cancer				
Negative test	4,595,260	9229	Ref.	Ref.
False-positive result	360,439	1308	1.97 (1.86–2.09)	2.04 (1.93–2.16)
Second false-positive result	15,186	86	3.62 (2.92–4.47)	4.71 (3.81–5.83)
Interval breast cancer				
Negative test	6,630,989	4815	Ref.	Ref.
False-positive result	569,334	826	2.06 (1.92–2.22)	2.18 (2.02–2.34)
Second false-positive result	30,072	59	2.92 (2.26–3.78)	4.22 (3.27–5.46)

^a^Hazard ratios from partly conditional Cox proportional hazards model were adjusted by age at screen (continuous), type of attendance (regular or irregular), mammography type (SFM or FFDM), and country (random effect)

**Fig. 1:**
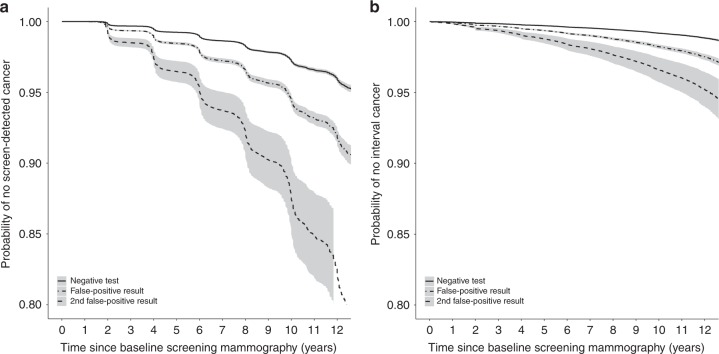
Adjusted survival curves for screen-detected and interval breast cancer based on Cox proportional hazards model for women with false-positive screening results vs women with negative screening tests. Models are adjusted for age at screen, type of attendance, mammography type, and country. Solid line represents negative screening mammogram group; dashed line represents false-positive screening result group; dotted line represents second false-positive screening result group. Fig. 1a represents screen-detected cancer; Fig. 1b represents interval breast cancer

We examined the adjusted survival curves for screen-detected and interval breast cancer (Fig. 1). The figure depicts how occurrence of screen-detected cancer follows a stagger 2-year pattern given by the biennial screening participations of women in the programs, whereas the risk for interval cancers follows a continuous pattern over time, since interval breast cancers can be diagnosed at any point of time in the 2-year interval after the index mammogram. The probability of remaining free of screen-detected cancer at 10 years for women with a negative test result was 97.2% compared to 94.4% for women with a first false-positive result and 87.5% for women with a second false-positive result (Fig. 1a). For interval breast cancers, the probability of remaining free of interval breast cancer at 10 years for women with a negative test result was 99.2% compared to 98.2% for women with a first false-positive result and 96.6% for women with a second false-positive result (Fig. 1b). Among those with a second false-positive result, the risk of developing breast cancer continued to diverge over time for both screen-detected and interval breast cancer.

Sensitivity analyses showed very little impact of the heterogeneity across countries in the estimates (Supplementary material, section 2). Also, the sensitivity analyses excluding breast cancers diagnosed in the first 26 months of follow-up were consistent with the estimates in the full model (Supplementary material, section 3).

## Discussion

In this joint analysis of individual level data from three population-based screening programs in Europe, we identified a two-fold higher risk of screen-detected and interval breast cancer among women with prior false-positive results compared to those with negative tests. The risk similarly increased four-fold after a second false-positive screening result. We also found that the risk for both screen-detected and interval breast cancer remained increased 12 years after experiencing the false-positive result.

Our findings are consistent with previous studies that analyzed long-term breast cancer risk after a false-positive result.^6–10,21^ A study from United States found that women with a false-positive screening result were at an increased risk of developing breast cancer for at least a decade.^6^ The risk estimates were somehow lower compared to our study, to HR of breast cancer diagnosis of 1.39 for women with a false-positive with additional imaging recommendation, and to HR = 1.76 for women with a biopsy recommendation, respectively. It is expected that the association between false-positive results and breast cancer risk is weaker in the US where mammography screening is opportunistic and mostly annual, compared with Europe where most countries perform biennial population-based screening. False-positive rates are substantially higher in the United States than in Europe,^5,11,22,23^ as evidenced by the much lower positive predictive value of recall.^24^ High false-positive rates are likely to underestimate the association between false-positive results and breast cancer risk compared with European countries where recall for further assessments is more selective. In the context of population-based screening in Europe, most prior studies analyzing the long-term risk of breast cancer after a false-positive result involve data partially included in our joint analysis. A study from Denmark found a 67% increased risk of breast cancer among women with prior false-positive results,^9^ whereas a study from Spain reported the odds ratio of subsequent screen-detected cancer to be 1.81.^8^ Also, on a previous joint study from Denmark, Norway, and Spain we reported a two-fold risk of screen-detected cancer in women with a false-positive result, and the risk remained significantly increased 6 or more years after the false-positive.^11^ In contrast with most findings, a study from the Netherlands including 188 women reported no excess breast cancer risk among women with a prior false-positive in a mean follow-up of 7 years, which is likely to be affected by the small sample size.^21^ A recent study from Norway found a higher risk for interval breast cancer in women with false-positive results than in our study (OR = 3.3).^25^ The study aimed at comparing tumour characteristics between interval breast cancers diagnosed in women with and without prior false-positive results. The authors did not include time as an adjusting variable in their analyses and the unit of analysis was the screening mammogram rather than the women. Consequently, their results are not comparable with our study.

Several studies have estimated the short-term risk of breast cancer in the screen subsequent to a prevalent false-positive screening result. In the context of population-based screening programs in Europe, a study from the United Kingdom found and increased risk of interval breast cancer and of screen-detected cancer at second screen in women with a false-positive result.^10^ An increased risk of breast cancer has also been reported in opportunistic screening in the US, where Barlow et al.^7^ found a higher risk of breast cancer (OR = 1.69) within 1 year after a false-positive result.

Misclassification is an unavoidable part of studies assessing the risk of breast cancer after a false-positive result. A study from Denmark showed that one out of every four women who later developed breast cancer after a false-positive result were misclassified at baseline assessment.^13^ Besides, the study also found that the average time from false-positive result to breast cancer diagnosis was significantly shorter among misclassified cases than among those non-misclassified (4.0 vs 7.8 years). The differences in average time are due to the fact that the cancer was already present at the time of false-positive result in misclassified cases. Another European study argued that three fourths of breast cancers diagnosed in women with false-positive result were already present at the baseline referral.^12^ They also showed that 63% of misclassified cases turned out to have breast cancer within 2 years after the false-positive result, while the proportion was 37% for cases in the reference group. It is thus likely that short-term studies are overestimated due to misclassification and lack of thorough follow-up time. Sensitivity analyses showed consistent results in our study population when breast cancers diagnosed in the first 26 months of follow-up were excluded from the analyses (Supplementary material, section 3). We also found that the time to diagnosis for screen-detected and interval breast cancers expanded for over 12 years after experiencing a negative test or false-positive result (Supplementary material, section 5).

The sustained long-term increased risk strengthens the idea of a biological susceptibility for developing future breast cancer in women with mammographic abnormalities. The results suggest that the mammographic abnormalities might be risk markers rather than precursors of subsequent breast cancer. This is consistent with the excess breast cancer risk found in women with a benign breast disease.^26–28^ In a previous study that used data partially included in this study, we found that women with a non-proliferative breast disease had a two-fold risk of breast cancer, whereas women with a proliferative breast disease had a three-fold risk, and women with a proliferative disease with atypia had over a four-fold risk.^26^ Interestingly, we also found that between 40 and 45% of breast cancer cases were contralateral to the prior benign breast lesion, which has been shown in other previous studies.^26,27^ The findings suggest that a large percentage of benign lesions, particularly those with a lower risk such as non-proliferative lesions and proliferative lesions without atypia, are a marker of future breast cancer risk rather than a pre-malignant lesion that will develop into a breast cancer.

In our study population, the screening programs share very similar standards and management policies. However, the number of women analyzed differed across countries, as well as the rates of screen-detected cancer, interval breast cancer, and false-positive results (Supplementary material, section 1). Variation across countries in the crude rates is expected, and the differences might be attributable to variations in clinical practice and in the characteristics of the populations.^29–31^ Nevertheless, the sensitivity analyses to test the impact of the heterogeneity across countries showed little impact on the overall estimates (Supplementary material, section 2), indicating that the observed effect of false-positive results on the risk of breast cancer is consistent in our joint analyses, independently of the inter-country variability in the rates.

We analyzed the risk of screen-detected cancer and interval breast cancer in women with false-positive result using individual level data from three population-based screening programs in Europe. The analyses are based on long-term follow-up data, which includes information from the start of each screening program, with a follow-up of more than 15 years. The availability of thorough data of each woman enabled us to map exposure and outcome on an individual level, with no loss to follow-up. However, the study has some limitations. Firstly, we lacked information on which recalls for further assessments lead to a biopsy recommendation, which would have added interesting information to estimate the risk associated with false-positive results including an invasive procedure with benign outcome. Previous studies have shown that the breast cancer risk increased after a biopsy recommendation compared to those with only additional imaging procedures.^6,9,25^ Secondly, we had no information on breast density which would have enriched our analyses. High mammographic density is a major risk factor for breast cancer,^32–34^ and has been also associated with an increased risk of false-positive results.^35^ Nevertheless, a previous study showed that mammographic density and prior false-positive results were independent risk factors for breast cancer, which minimises the possible bias introduced by this lack of information. Finally, we had no information on laterality of the mammographic finding that caused the recall for further assessments and thus, we are unable to assess how many cancers developed at the site of the initial mammographic lesion.

No prior studies have assessed differences in the long-term risk of screen-detected cancer and interval breast cancer after a first and second false-positive result in population-based mammography screening. We observed a similarly increased risk of screen-detected and interval breast cancer among women with a false-positive result. Consistently, the risk similarly increased four-fold after a second false-positive result. Interval breast cancers are a major detriment of mammography screening. Women with an interval breast cancer diagnosis do not have a substantial benefit from routine screening participation. Based on our findings it might be advisable to inform women with false-positive results about their specific increased risk for screen-detected and interval breast cancer. The highly increased risk reveals a sub-group of women that may be eligible for more intensive screening strategies. Since false-positive results affect a large number of screened women, the outcome of previous screening participations should be considered in risk prediction models aimed at personalizing breast cancer screening strategies based on women’s individual breast cancer risk.

In conclusion, we found that women with false-positive results had a two-fold risk of having a later screen-detected and interval breast cancer. The risk increased to a four-fold after a second false-positive screening result and remained elevated for 12 years after experiencing the false-positive result.

### Availability of data and materials

The datasets generated and analysed during the current study are not publicly available, but are available from the authors on reasonable request.

## Supplementary information

Supplementary Material
