# Supplementary material for: Long-term risk of screen-detected and interval breast cancer after false-positive results at mammography screening: joint analysis of three national cohorts
Source: Br J Cancer. 2018 Dec 19;120(2):269–75. doi: 10.1038/s41416-018-0358-5 (PMC6342908; doi:10.1038/s41416-018-0358-5)

**Long-term risk of screen-detected and interval breast cancer after false-positive results at mammography screening: Joint analysis of three national cohorts**

Marta Román, Solveig Hofvind, My von Euler-Chelpin, and Xavier Castells

## Supplementary material

1. **Descriptive table of the characteristics of the study population by country.**

1. **Sensitivity analyses to test the impact of the heterogeneity across countries**

The sensitivity analysis showed very little impact of the heterogeneity across countries in the estimates. Compared with the estimates of the fully adjusted model (including country as a random effect) in Table 4, the estimates of the adjusted model excluding the country effect were HR_False-positive_ =2.01 (95%CI: 1.90-2.13) and HR_2nd false-positive_= 4.48 (95% CI: 3.62-5.54) for screen-detected cancer and HR_False-positive_ =2.13 (95%CI: 1.98-2.29) and HR_2nd false-positive_ =3.91 (95%CI: 3.02-5.06) for interval breast cancer. In the adjusted model including country as a fixed effect, the estimates were HR_False-positive_ =2.04 (95%CI: 1.93-2.16) and HR_2nd false-positive_=4.73 (95%CI: 3.82-5.85) for screen-detected cancer and HR_False-positive_ =2.18 (95%CI: 2.02-2.34) and HR_2nd false-positive_=4.22 (95%CI: 3.26-5.46).

1. **Sensitivity analyses to test the impact of misclassification**

1. **Effect of adjusting variables in the model estimates.**

1. **Time to diagnosis of screen-detected cancers and interval breast cancers by screening mammogram result.**

**Additional figure 1, section 5**: Time to diagnosis of screen-detected cancers by screening mammogram result.


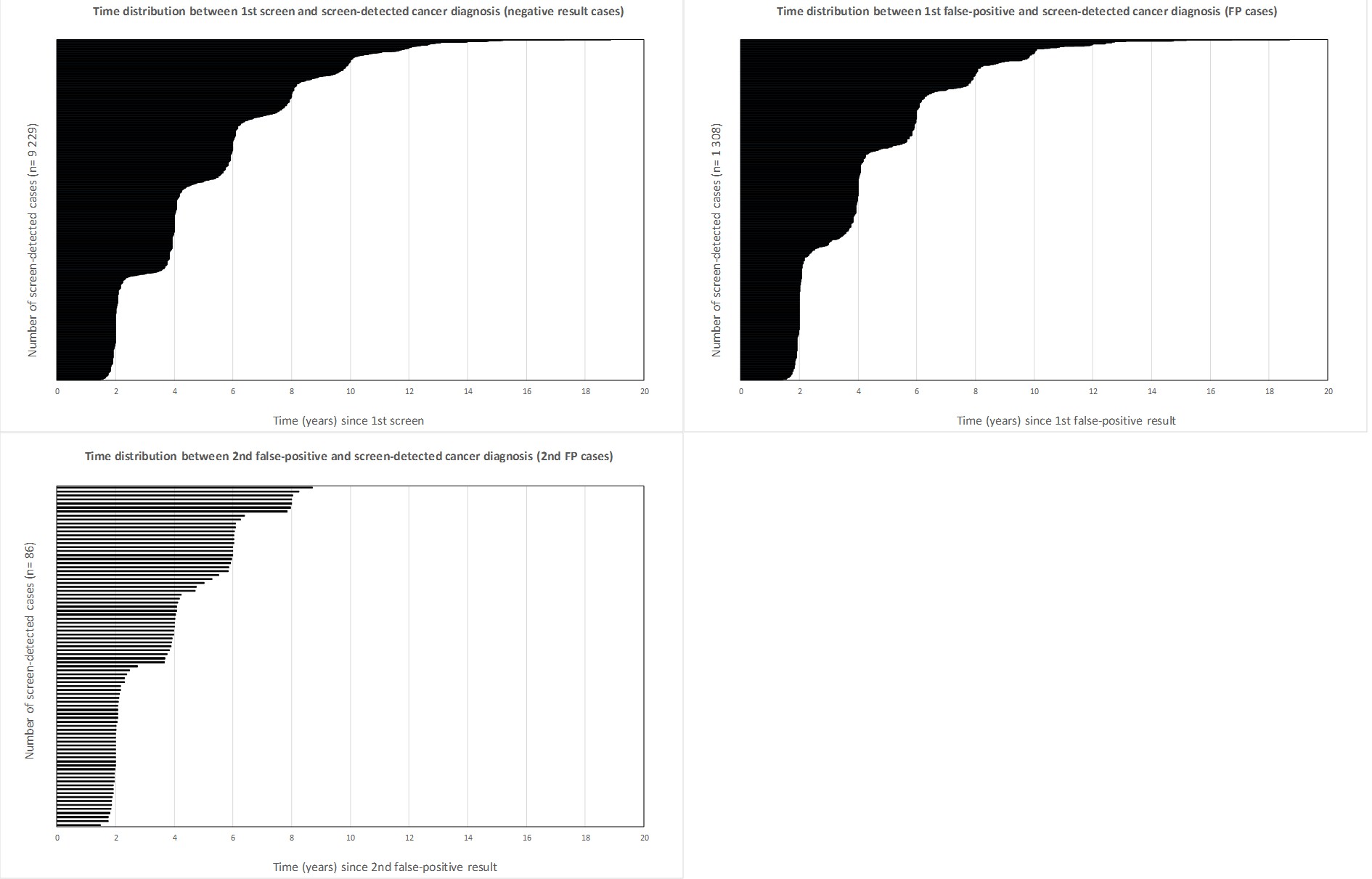


**Additional figure 2, section 5**: Time to diagnosis of interval breast cancers by screening mammogram result.


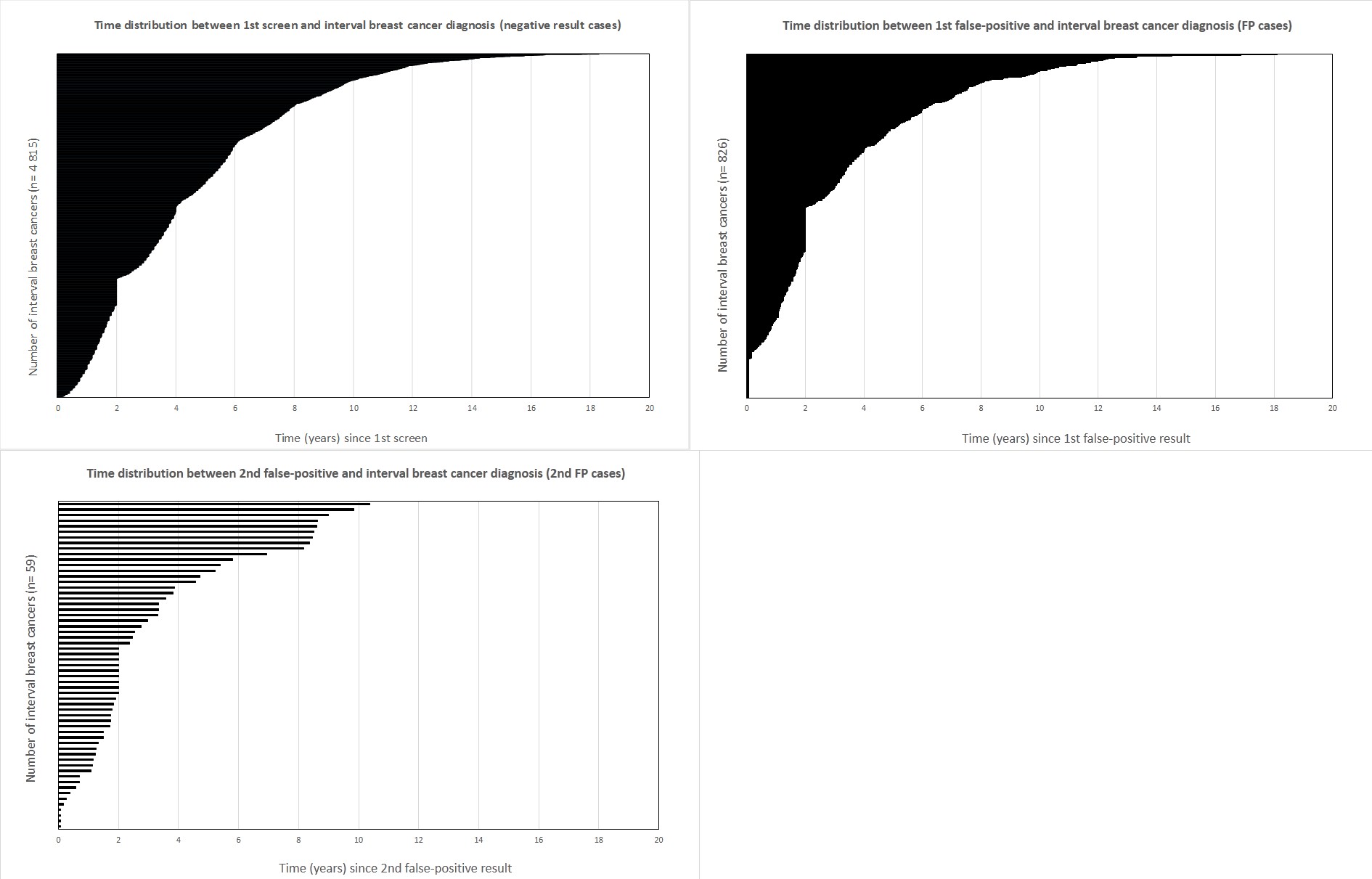

Supplement: Supplementary file 1 — Supplementary Material [file 41416_2018_358_MOESM1_ESM.docx]
